# Anterior coverage after eccentric rotational acetabular osteotomy for the treatment of developmental dysplasia of the hip

**DOI:** 10.1007/s00776-014-0592-5

**Published:** 2014-06-23

**Authors:** Hiroshi Imai, Tomomi Kamada, Jun Takeba, Yoshitaka Shiraishi, Naohiko Mashima, Hiromasa Miura

**Affiliations:** Department of Bone and Joint Surgery, Ehime University Graduate School of Medicine, Shitsukawa, Toon, Ehime 791-0295 Japan

## Abstract

**Background:**

In periacetabular osteotomy for the treatment of developmental dysplasia of the hip, impairments in ADL due to limitations in hip flexion can occur when anterior displacement is added to lateral displacement in order to obtain sufficient femoral head coverage. This study was conducted to determine, by the range of motion (ROM) simulation based on CT images, the minimum angle of hip flexion and internal rotation at 90° of flexion that is necessary to avoid ADL impairments after eccentric rotational acetabular osteotomy (ERAO) and to estimate the angles of anterior femoral head coverage on plain radiography that enable the above flexion.

**Methods:**

Of 47 hips treated with ERAO at our hospital from December 2007 to May 2012, 27 hips without progressive osteoarthritis which could be CT scanned were examined and included. The mean age at the time of surgery was 40.7 years (SD 1.8). The postoperative follow-up period was 30.2 months (SD 3.6). Two hips were in male patients and 25 hips were in female patients. The disease stage prior to surgery was pre-osteoarthritis in 5 hips, early in 11 hips, and progressive in 11 hips. We checked whether the patients were capable of activities that require deep hip flexion for the evaluation of postoperative ADL. Radiographic examination was performed before and one year after surgery to calculate LCE angle, Sharp angle, AHI, and VCA angle. The angle at which impingement of the displaced fragment of the bone and the femur appeared was measured using 3D CAD software, and the relationship between this angle and the physical findings, ADL impairment, or radiographic findings, were also examined.

**Results:**

22 out of 27 hips that were capable of 116° or more of flexion or 42° or more of internal rotation at 90° of flexion in ROM simulation showed the absence of ADL impairment and a postoperative VCA angle ≤42°, whereas 5 hips with 110° or less of flexion or 40° or less of internal rotation at 90° of flexion in ROM simulation had ADL impairments associated with limitations in hip flexion and a postoperative VCA angle ≥46°.

**Conclusions:**

Anterior and lateral coverage requires a postoperative VCA angle of ≥20° to achieve anterior structural stability and an LCE angle of >25° to obtain adequate superior lateral coverage of the femoral head. A VCA angle ≤42° is required to avoid impingement during deep flexion. A VCA angle ≥46° is a probable risk factor for pincer FAI syndrome after ERAO.

## Introduction

The important factors for successful periacetabular osteotomy such as rotational acetabular osteotomy (RAO) [1], eccentric rotational acetabular osteotomy (ERAO) [2], the Bernese periacetabular osteotomy (PAO) [3], and curved periacetabular osteotomy (CPO) [4], and therapeutic methods for developmental dysplasia of the hips (DDH) are: (1) adherence to the indications of periacetabular osteotomy, for example, cases where the curvature of the acetabulum matches that of the femoral head as observed in X-ray images [1, 2, 4, 5], or with a remaining joint space of 2 mm or larger [6]; (2) technical avoidance of intrusion of the chisel into the joint; and (3) sufficient coverage of the femoral head with the displaced fragment of acetabular roof. However, when anterior displacement is added to lateral displacement in order to obtain sufficient coverage of the femoral head, activities of daily living (ADL) impairment due to limitations in the range of motion (ROM) of the hip joint and pincer femoroacetabular impingement (pincer FAI) syndrome [7] can occur.

The objective of this study was to determine, by evaluation of ROM with physical examination and the computer simulation, the minimum angle of hip flexion that is necessary to avoid impairments in ADLs after ERAO for the treatment of symptomatic DDH and to estimate the angles of lateral or anterior femoral head coverage on plain radiography that enable the above flexion.

## Materials and methods

**Fig. 1:**
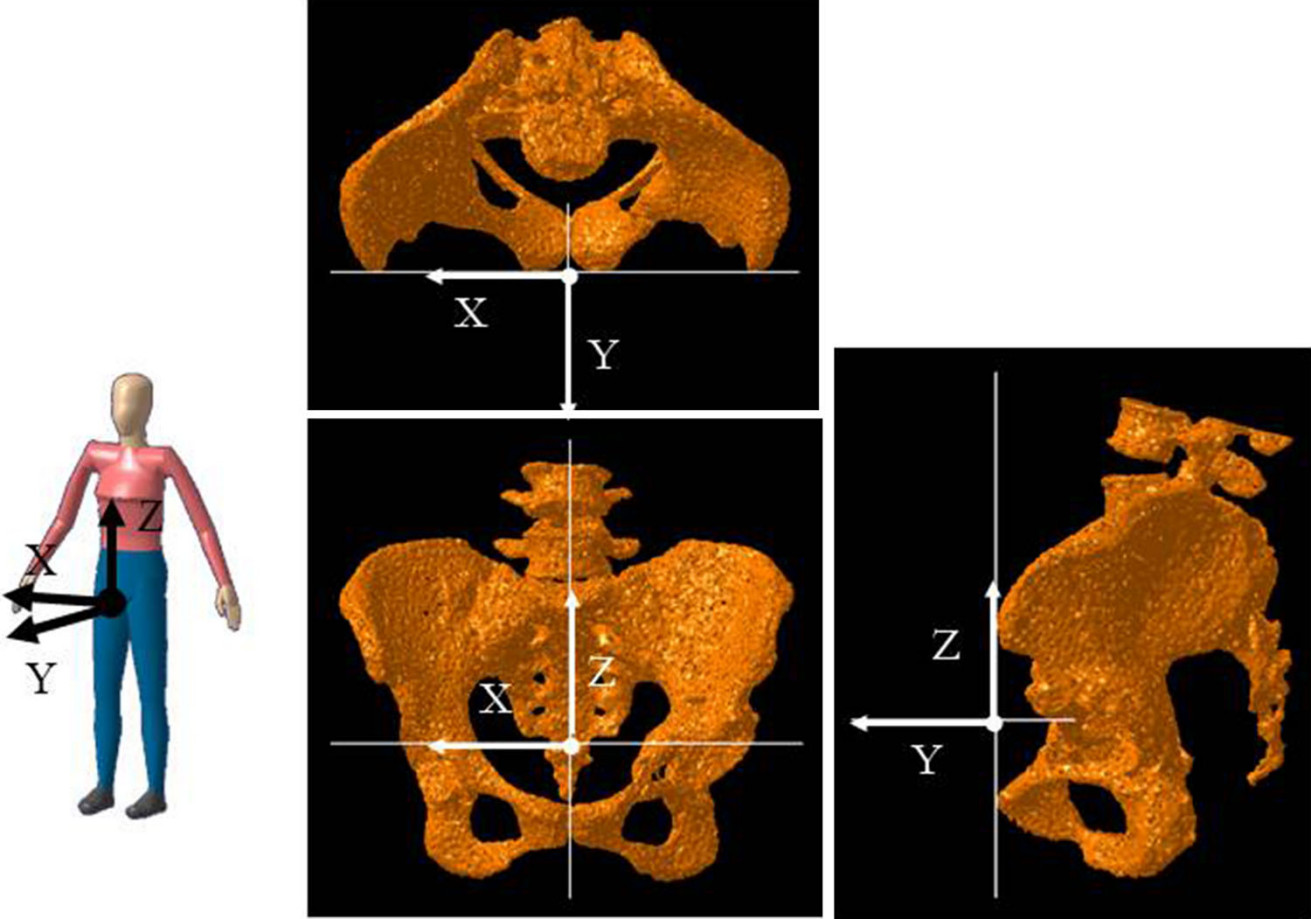
Definition of pelvic reference coordinate system

**Fig. 2:**
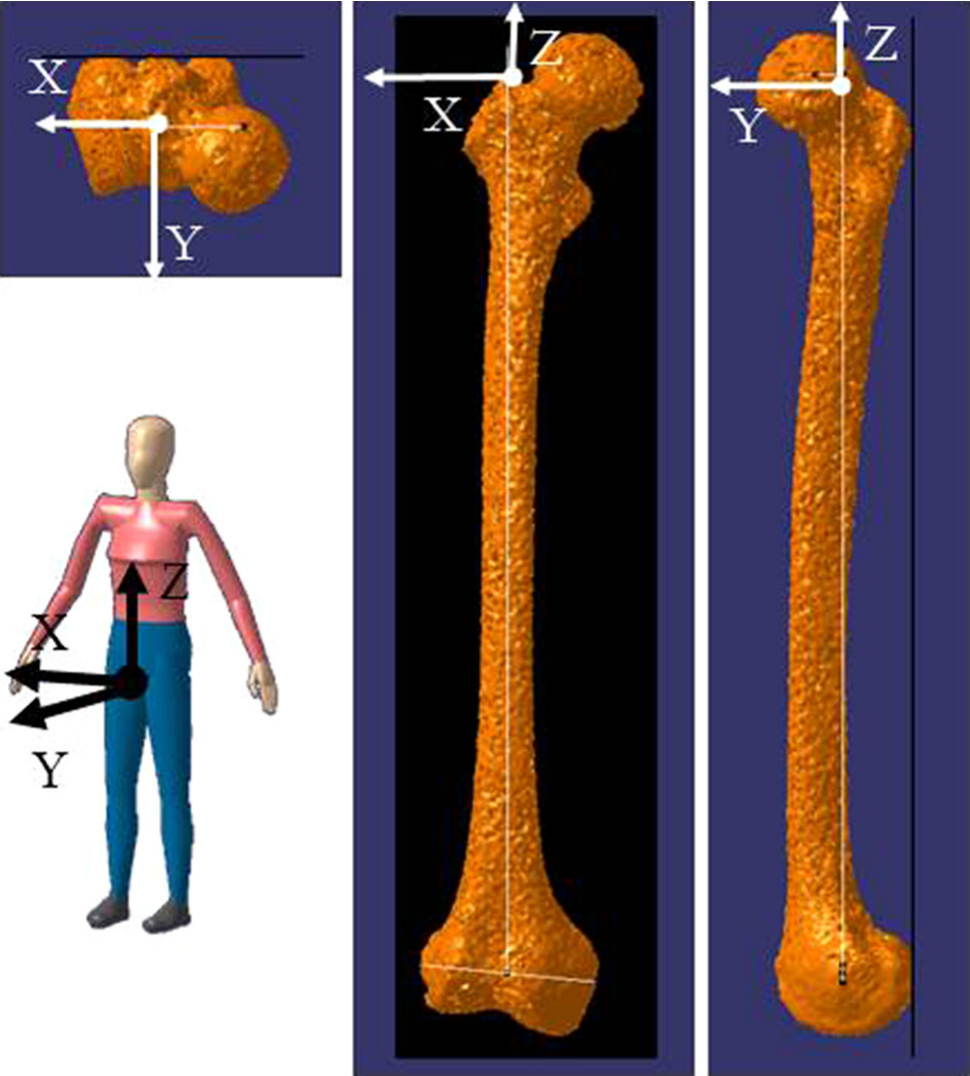
Definition of femoral reference coordinate system

This study was approved by our institution’s scientific research board, and it was conducted in accordance with the World Medical Association Declaration of Helsinki of 1964 as revised in 1983 and 2000. All patients were informed about the study in detail and provided written informed consent before their enrollment, including consent to the acquisition of the postoperative computer tomography. Of 47 hips treated with ERAO at our hospital from December 2007 to May 2012, 27 hips without progressive osteoarthritis which could be CT scanned were examined and included. Twenty hips in total were excluded: 16 that had end-stage osteoarthritis as discovered during post-operative follow-up examinations and 4 that could not be examined by CT scan. The mean age of the patients at the time of surgery was 40.7 years (SD 1.8 years, range 25–55 years). The postoperative follow-up period was 30.2 months (SD 3.6 months, range 12–68 months). Two hips were in male patients and 25 hips were in female patients. Mean BMI was 22.6 kg/m^2^ (SD 0.7 kg/m^2^, range 16.7–32.2 kg/m^2^). The disease stage prior to surgery was pre-osteoarthritis in 5 hips, early osteoarthritis in 11 hips, and progressive osteoarthritis in 11 hips [1]. The examination items included the Japanese Orthopaedic Association (JOA) hip score before and at each follow-up after osteotomy [8], the presence or absence of anterior thigh pain in deep hip flexion, and anterior impingement signs [9]. The JOA hip score was used to assess the subjective parameters of pain (0–40 points), walking ability (0–20 points), range of motion (0–20 points), and function (0–20 points). The physical hip flexion angle was measured with the patient supine and with the contralateral lower extremity fixed to the table with 0° of rotation in both lower extremities to prevent pelvic extension compensation. This was done twice by two orthopedic surgeons (HI and TK) with more than 15 years of experience. The time between measurements was at least 2 weeks. Intra- and inter-observer variances were calculated. We also checked whether the patients were capable of the following five activities that require deep hip flexion for the evaluation of postoperative ADL: (1) putting on and taking off socks, (2) clipping toenails, (3) tying shoelaces, (4) using a Japanese-style toilet, and (5) sitting on a low chair [9, 10]. Radiographic examination was performed before and one year after surgery to calculate the lateral center edge (LCE) angle [11], Sharp angle [12], acetabular-head index (AHI), the vertical axis-center of the femoral head, the anterior extremity of the acetabular roof (VCA) angle in the false profile view [13], the acetabular version, and the posterior wall sign. The angle at which impingement of the displaced fragment of the bone and the femur appeared was measured using three-dimensional computer-aided design (3D CAD) software [14], and the relationships between this angle and the above physical findings, ADL impairment, or radiographic findings were also examined. The pelvic coordinate system and the femoral coordinate system were determined referring to Cappozzo et al.’s report [15]. In determining the pelvic coordinate system, a plane that includes the two most anterior points of right and left anterior superior iliac spines and the two most anterior points of the pubic bone was defined as the *XZ* plane, the axis connecting the two most anterior points of right and left anterior superior iliac spines as the *X* axis, the axis on the *XZ* plane that was perpendicular to the *X* axis as the *Z* axis, and the cross-product of the *X* axis and *Z* axis as the *Y* axis (Fig. 1). For the femoral coordinate system, a plane that includes the most posterior point of the femur on the proximal side and medial and lateral posterior femoral condyle points was formed first. Next, the femoral axis connecting the piriformis fossa and the point midway between the medial and lateral condyles was formed. Then, this femoral axis was shifted using the piriformis fossa as a center of rotation so that the axis became parallel to the plane formed as described above. The resulting axis was defined as the *Z* axis, the axis going through the piriformis fossa perpendicular to the *Z* axis and parallel to the formed plane as the *X* axis, and the cross-product of the *X* axis and *Z* axis as the *Y* axis (Fig. 2). Because hip ROM was evaluated using the femoral head as a virtual center of rotation, the piriformis fossa was set as the center for defining the coordinate system. This was shifted to the center of the femoral head in creating hip flexion. Hip flexion and a combination of flexion and internal rotation were simulated, and the angle causing impingement was determined by calculating the overlapping area as the area in contact. The measurement of the angle causing impingement was corrected for the tilting angle of the pelvis to the surface of the bed [16]. All radiographic measurements were reported by the same observer (HI).

### Operative technique

The eccentric rotational acetabular osteotomy was performed according to the technique described by Hasegawa [2]. The patient is positioned in the lateral position. The greater trochanter is detached with an oscillating saw and is reflected proximally. A curved osteotomy chisel is introduced proximately 15 mm superior to the joint space, and an eccentric osteotomy is made. All acetabular osteotomies were performed with a curved 45 mm-radius chisel. The angle and direction of the osteotomy are determined with an intraoperative X-ray. Rotation of the acetabular fragment allows medial and distal displacement of the femoral head to be obtained simultaneously.

The osteotomized acetabular fragment is moved laterally and about 1 cm anteriorly to obtain superior lateral and anterior coverage of the femoral head. An LCE angle of >25° is necessary to obtain adequate superior lateral coverage of the femoral head, and a VCA angle of ≥20° is needed to achieve anterior structural stability [17]. After the fragment of the acetabular roof is displaced, 3–4 hydroxyapatite fixation screws were used for fixation of the acetabular fragment.

### Scanning procedure and measurement

The 3D-CT scans were performed using a Philips Brilliance 64 scanner (Marconi medical System, The Netherlands). The scanning technique used was 120 kV, 150–250 effective mAs (depending on the patient’s size), with a 0.5 s rotation time. Contiguous slices (2.0 mm) were obtained from the bilateral anterior superior iliac spines to the femoral distal end, with the patients placed in a supine position with hips extended and thighs horizontal and parallel. The images were reconstructed at the CT workstation (DELL PRECISION T7600) to produce the 3D images (Microsoft Visual studio 2008).

### Statistical analysis

The normality of the continuous data was checked with the Kolmogorov–Smirnov test. Therefore, because of the data’s normal distribution, an unpaired Student’s *t* test was used for comparison of normally distributed continuous data. SPSS for Windows version 20 (SPSS Inc., Chicago, Illinois) was used for all statistical analyses. Statistical significance was set at a value of *p* < 0.05. Intra-observer variance in hip flexion angle was determined by comparing separate assessments of the same patient by the same observer with at least a 2-week intermission between assessments. Intra- and inter-observer variances in hip flexion angle were expressed using interclass correlation coefficients (ICC) with: ICC <0.20 for slight agreement; 0.21–0.40 for fair agreement; 0.41–0.60 for moderate agreement; 0.61–0.80 for substantial agreement; and >0.80 for almost perfect agreement [18].

## Results

The mean JOA hip score was 67.8 points (SD 2.3 points; range, 43–89 points) prior to surgery and 88.1 points (SD 1.4 points; range, 79–97 points) after surgery, showing a significant improvement (*p* < 0.01). Two intra-observer interclass correlation coefficients (ICCs) were calculated, both at 0.99. Inter-observer variance had an ICC of 0.98. These indicate almost perfect agreement on hip flexion angle as measured in physical examinations. The mean angle of flexion was 110.1° (SD 2.1°; range, 90°–135°) in the physical examination pre-operatively and 105.8° (SD 2.6°; range, 80°–120°) at the last follow-up visit after the surgery; 5 hips were 90° or less, and 22 hips were capable of 100° or more of flexion at the last follow-up visit. Anterior thigh pain in deep hip flexion was reported in 3 of 5 hips, with 90° or less of hip flexion angle at the last follow-up visit after surgery. The anterior impingement sign that was not noted in pre-operative physical examination appeared in all 5 hips with a 90° or less flexion angle following surgery. In radiographic examinations, the LCE angle, Sharp angle, AHI, and the VCA angle were 4.2° (SD 2.2°; range, 16°–22°), 49.4° (SD 0.7°; range, 42°–56°), 54.2 % (SD 2.2 %; range, 34.6–72.1 %), and 21.9° (SD 2.1°; range, 4°–39°), respectively, prior to surgery, and 34.2° (SD 1.6°; range, 11°–49°), 36.9° (SD 0.5°; range, 33°–42°), 82.7 % (SD 1.5 %; range, 60.3–92.8 %), and 36.3° (SD 3.2°; range, 13.0°–70.0°), respectively, after surgery, indicating significant improvement (*p* < 0.01, *p* < 0.01, *p* < 0.01, *p* < 0.01, respectively) (Table 1). Acetabular versions were anteversion in 24 hips, retroversion in 3 hips prior to surgery. After surgery, acetabular versions were changed from anteversion to retroversion in 2 hips, from retroversion to anteversion in 1 hip; 2 hips remained in retroversion, and 22 remained in anteversion. Posterior wall sign values were positive in 2 hips and negative in 25 hips prior to surgery. After surgery, posterior wall sign values changed from positive to negative in 1 hip; 1 hip remained positive, and 25 hips remained negative. Regarding activities of ADL, surgeries in 5 hips resulted in difficulty or inability to tie shoelaces. However, use of a Japanese-style toilet and a low chair was still feasible. All five activities could be carried out post-operatively in the remaining 22 hips. 5 hips that with a 90° or less flexion angle in physical examinations at the last follow-up visit after the surgery all have the presence of ADL impairment. The angle at which impingement occurred in the ROM simulation based on CT images was calculated. The mean angle of flexion and internal rotation at 90° of flexion were 120.0° (SD 3.2°; range, 87°–163°) and 44.4° (SD 4.6°; range, 18° to 83°), respectively, in the ROM simulation. The angle at which impingement occurred in the ROM simulation was compared in the presence or absence of ADL impairment, flexion angle, and internal rotation at 90° of flexion were 102.6° (SD 2.2°; range, 87°–110°) and 21.6° (SD 5.5°; range, 18°–40°), respectively in five hips with a 90° or less flexion angle in physical examinations with impairments in ADLs. In these five cases, impingement occurred at flexion angles of 87°, 103°, 104°, 109°, 110°, respectively, and at internal rotation at 90° flexion angles of −18°, 23°, 29°, 34°, 40°, respectively. The case with a flexion angle of 87° was due to excessive anterior femoral coverage and resulted in ADL impairment. However this case was not progressive after the osteotomy for 4 years.

**Table 1:** Radiographic results

	Preoperative	Postoperative	*p* value
LCE angle (°)^a^	4.2 ± 2.2	34.2 ± 1.6	<0.01
	(−16 to 22)	(11–49)	
Sharp angle (°)^a^	49.4 ± 0.7	36.9 ± 0.5	<0.01
	(42–56)	(33–42)	
AHI (%)^a^	54.2 ± 2.2	82.7 ± 1.5	<0.01
	(34.6–72.1)	(60.3–92.8)	
VCA angle (°)^a^	21.9 ± 2.1	36.3 ± 3.2	<0.01
	(4.0–39.0)	(13.0–72.0)	

^a^Values are expressed as mean ± SD; with the range in parentheses

We observed 129.3° (SD 2.8°; range, 116°–163°) of flexion and 58.1° (SD 2.9°; range, 42°–83°) of internal rotation at 90° of flexion in 22 hips capable of 100° or more of the flexion without impairment in ADLs. The range of motion in hip flexion and internal rotation at 90° of flexion in ROM simulation were thus significantly greater in the group without impaired ADLs (*p* < 0.01, *p* < 0.01, respectively). All cases of impingement at 110° of flexion or less in ROM simulations impinged when the angle of flexion was 40° or less of internal rotation at 90° of flexion, and all cases of impingement at 116° of flexion or more did not impinge when the angle of flexion was 42° or more of internal rotation at 90° of flexion. Considering the above result, postoperative radiographic findings of the group showing impingement at 90° or less of flexion in physical examination (*n* = 5) and those of the group that was capable of 100° or more of flexion (*n* = 22) were compared. The postoperative LCE angle of the former group and the latter group was 36.0° (SD 4.5°; range, 25°–49°) and 33.8° (SD 1.7°; range, 11°–43°), respectively. The Sharp angles of these groups were 36.6° (SD 1.0°; range, 34°–40°) and 37.0° (SD 0.6°; range, 33°–42°), respectively; AHI was 83.0 % (SD 4.4 %; range, 70.2–92.8 %) and 82.6 % (SD 1.6 %; range, 60.3–90.0 %), respectively. The VCA angle was 54.4° (SD 4.2°; range, 46.0°–72.0°) and 32.1° (SD 1.8°; range, 13.0°–42.0°), respectively. There was a significant difference in the VCA angle (*p* < 0.01), but no significant difference was observed between these groups in the LCE angle, Sharp angle, and AHI (*p* = 0.6, *p* = 0.8, *p* = 0.9, respectively) (Table 2). The postoperative acetabular versions of the former group and the latter group were retroversion in 3 of 5 hips and 1 of 22 hips, respectively.

**Table 2:** Comparison in the presence or absence of impingement in physical examination

	Impingement + (*n* = 5)	Impingement − (*n* = 22)	*p* value
Flex. in ROM simulation (°)^a^	102.6 ± 2.2	129.3 ± 2.8	*p* < 0.01
	(87–110)	(116–163)	
Int. rot. at 90° of flex. in ROM simulation (°)^a^	21.6 ± 5.5	58.1 ± 2.9	*p* < 0.01
	(−18 to 40)	(42–83)	
LCE angle (°)^a^	36.0 ± 4.5	33.8 ± 1.7	0.6
	(25–49)	(11–43)	
Sharp angle (°)^a^	36.6 ± 1.0	37.0 ± 0.6	0.8
	(34–40)	(33–42)	
AHI (%)^a^	83.0 ± 4.4	82.6 ± 1.6	0.9
	(70.2–92.8)	(60.3–90.0)	
VCA angle (°)^a^	54.4 ± 4.2	32.1 ± 1.8	<0.001
	(46.0–72.0)	(13.0–42.0)	

^a^Values are expressed as mean ± SD, with range in parentheses

22 hips that were capable of 116° or more of flexion or 42° or more of internal rotation at 90° of flexion in ROM simulation showed the absence of ADL impairment and a postoperative VCA angle ≤42°, whereas 5 hips with 110° or less of flexion or 40° or less of internal rotation at 90° of flexion in ROM simulation had ADL impairments associated with limitations in hip flexion and a postoperative VCA angle ≥46°.

## Case

**Fig. 3:**
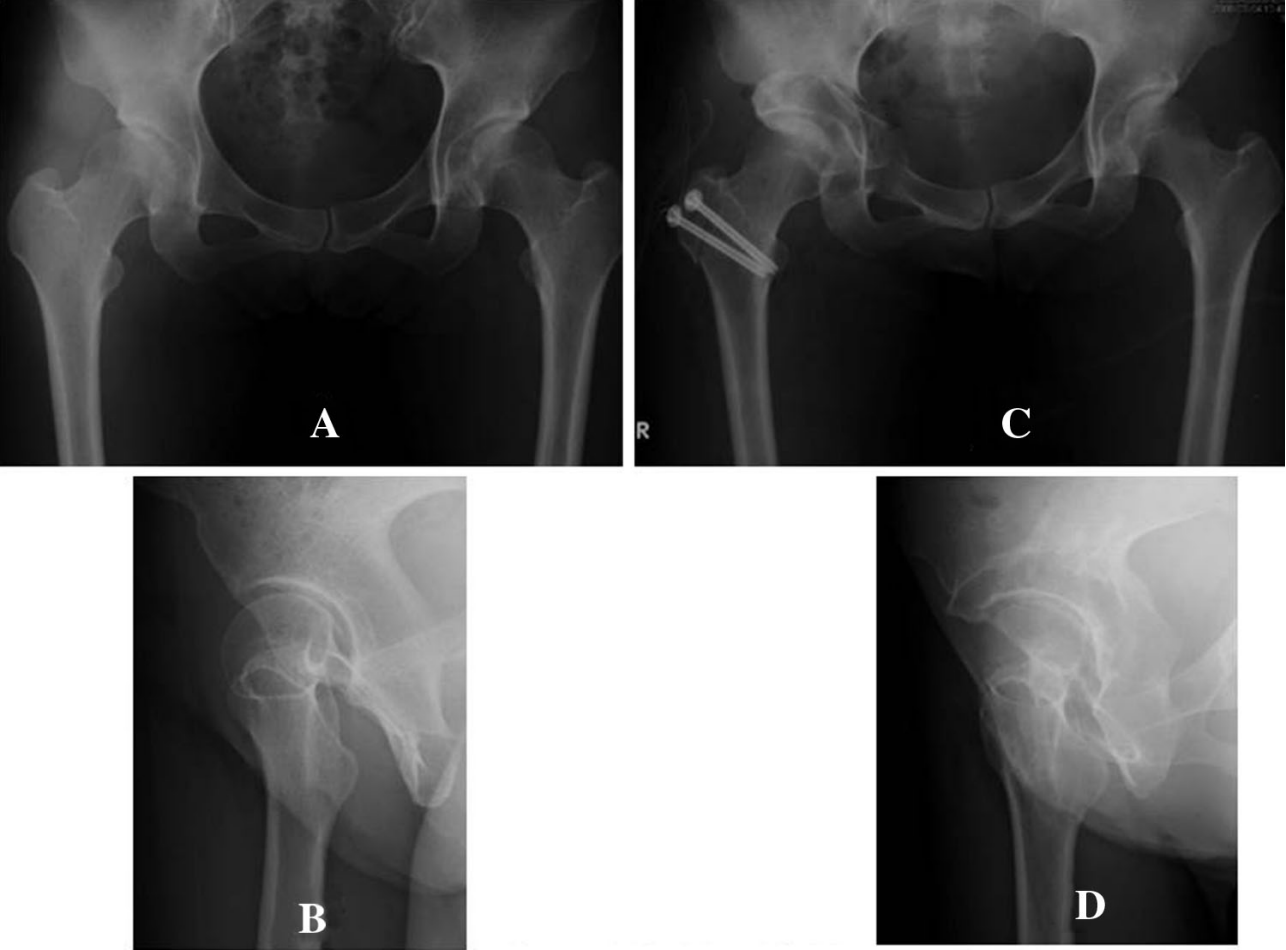
A right hip of a 36-year-old-female patient with developmental dysplasia of the hip. **a**, **b** On the conventional AP pelvic radiograph and the false profile view, the LCE and the VCA angle are 12° and 33°, respectively. **c**, **d** After ERAO, the LCE and the VCA angle are 33° and 53°, respectively

**Fig. 4:**
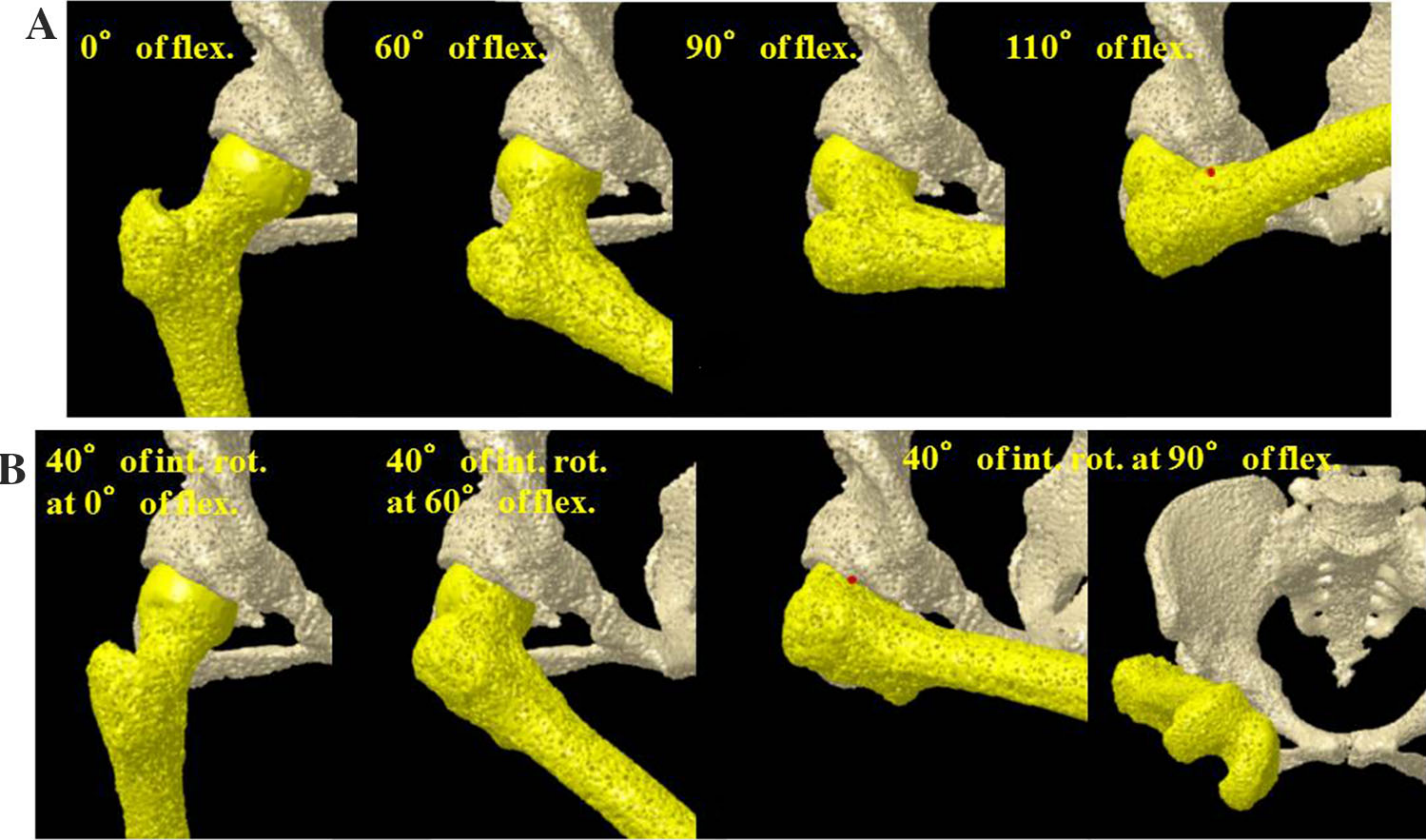
The ROM simulation based on postoperative CT images showed impingement of the displaced fragment of acetabular roof and the anterior surface of the femoral neck at a hip flexion angle of 110° (**a**) and 40° of internal rotation in 90° of flexion (**b**)

A 36-year-old woman without congenital dislocation of the hip had a chief complaint of pain in the right hip joint that started in March 2008 and was gradually aggravated. Due to the aggravation, ERAO of the right hip was performed in July 2008 (Fig. 3a–d). The JOA hip score was 66 points prior to surgery and was improved to 83 points one year after surgery. The angle of hip flexion was 110° in the physical examination pre-operatively and 90° at the last follow-up visit after the surgery. X-ray images showed that LCE angle, Sharp angle, AHI, and VCA angle had improved before and after surgery from 12° to 33°, from 49° to 36°, from 62.6 to 85.3 %, and from 33° to 53°, respectively. Acetabular version was changed from anteversion to retroversion. With regards to ADL, anterior thigh pain in walking up and down the stairs or in deep hip flexion remained after surgery, and it became difficult for her to tie shoelaces, use a Japanese-style toilet, and sit on a low chair. The ROM simulation based on postoperative CT images showed impingement of the displaced fragment of acetabular roof and the anterior surface of the femoral neck at a hip flexion angle of 110° and 40° of internal rotation at 90° of flexion (Fig. 4).

## Discussion

FAI is an osteochondral lesion caused by the collision of the acetabular edge and the femur. FAI is considered to contribute to primary osteoarthritis of the hip and is classified into two types. Cam type is caused by bony protrusion in the junction between the femoral head and the neck, and the other is pincer type, caused by excessive acetabular coverage. Cam type includes slipped capital femoral epiphysis, Perthes disease, avascular necrosis of the femoral head, coxa plana, and malunion of transcervical fractures [19–21]. Pincer type includes acetabular protrusion, acetabular retroversion, acetabular retroversion in Perthes disease, both after periacetabular fracture and after pelvic osteotomy [22–24]. Among treatment methods for developmental dysplasia of the hip, RAO [1, 5, 6], ERAO [2] and periacetabular osteotomy [3, 4] are performed by resecting the acetabulum surrounding the femoral head, including acetabular cartilage, and displacement of the resected fragment laterally, thereby sufficiently covering the femoral head in order to prevent the progression of disease [1–6]. However, Dong Hun Suh et al. [25] suggested that, in covering the femoral head, anterior displacement of the fragment of the bone is necessary in addition to lateral displacement in some cases, based on the virtual osteotomy using CT images of the hips with the acetabular dysplasia. Nevertheless, coverage of the femoral head by excessive anterior displacement may increase the incidence of postoperative FAI. Siebenrock et al. [26] reported that a pincer FAI occurred in 29 % of the cases they examined after periacetabular osteotomy. Since the introduction of the FAI concept, more emphasis was put on avoiding anterior and lateral overcorrection, which could be associated with an unfavorable outcome [27]. In our present study, ADL impairments were associated with limitations in the range of motion of hip flexion observed in five hips (18.5 %) after ERAO. In all five hips with ADL impairments, impingement occurred at a 90° or less the flexion angle in physical examinations at the last follow-up visit after the surgery and at 110° or smaller flexion angles or 40° or less of internal rotation at 90° of flexion to the functional pelvic plane in ROM simulation. In contrast, the hips in which impingement did not occur even at 100° or larger in physical examinations and at 116° or larger flexion or 42° or more of internal rotation at 90° of flexion in ROM simulation did not have ADL impairments. Hip flexion angle in physical examinations was about 20° less than flexion in ROM simulation due to soft tissue impingement.

In order to avoid hip flexion disturbance after ERAO and to obtain favorable long-term results,there should be pre-operative planning before ERAO for the treatment of symptomatic DDH and a ROM simulation after the virtual osteotomy with 3D CT. Anterior and lateral coverage requires a postoperative VCA angle of ≥20° to achieve anterior structural stability and an LCE angle of >25° to obtain adequate superior lateral coverage of the femoral head. A VCA angle ≤42° is required to avoid impingement during deep flexion (116° or more) or during 42° or more of internal rotation at 90° of flexion, as shown in the ROM simulation. A VCA angle ≥46° is a probable risk factor for pincer FAI syndrome after ERAO.

## Limitations

We did not consider that compensated pelvic extension frequently occurs in deep hip flexion in ROM simulation. Other problems in this study include a lack of consideration of the following factors in ROM simulation: (1) involvement of soft tissue, (2) proximal femoral head and neck deformity, (3) shift of the actual center of rotation because of the assumption that the femoral head is spherical and the definition of the center of the sphere as the center of rotation, and (4) width of the cartilage on the femoral head. Finally, our conclusions are limited due to the small number of cases (*n* = 27) in this report.

## Conclusions

We determined the minimum angle of hip flexion that is necessary to avoid impairments in ADLs after ERAO for the treatment of symptomatic DDH using ROM simulation and physical examination. Using plain radiography, we also estimated the angles of anterior femoral head coverage that enable the above flexion.

Anterior coverage requires a postoperative VCA angle ≥20° to achieve anterior structural stability. A VCA angle ≤42° is required to avoid impingement during deep flexion (116° or more) or during 42° or more of internal rotation at 90° of flexion, as shown in the ROM simulation.

A VCA angle ≥46° is a probable risk factor for pincer FAI syndrome after ERAO.
